# Indents

**Published:** 1922-08

**Authors:** 


### INDENTS

**☞Brief Articles of Technical or Scientific Value will receive notice and comment. Contributions invited.**

---

Practical Hints for Oxygen Gas Extractions. Do not use gas on very stout patients or you will have trouble. Use a local instead.

It is usually safe to use it on anyone who is well enough to walk to the office if properly administered.

It is essential that the patient have confidence in the operator; tell the patient to concentrate on a pleasant thought; do not give any instructions about breathing unless the patient starts to breathe too fast; if instructed beforehand he will surely drain the bag. If the gas tank goes dry when half under, the case will usually be a failure.

One of the chief reasons for failure to get the tooth and loss of confidence on the part of the patient is caused by the patient slipping down in the chair while under the gas. To prevent this occurrence all one has to do is to close in the arms of the chair against the thighs before starting and it will hold the patient as in a vise. The only way he can get up is to take the chair with him.

As a rule it is foolish to extract a third molar that is as thin as an egg shell as it is a dangerous procedure fishing out the pieces, and if the root is cone shaped it usually shoots out of the forcep. If this occurs drop the forceps, pull the head forward and get out the pieces with your fingers.

To prevent pieces of teeth passing down the throat adjust the patient's head so that the occlusal surfaces of the upper posterior teeth are practically level; this will cause the base of the tongue to rest against the soft palate thereby closing the throat from the mouth.

One of the dangers of using the nasal inhaler for extraction work is the possibility of giving the patient too much gas thus decreasing the reflexes. If one should lose a tooth in this condition the patient can not readily cough it up.

As a general rule it is better to do the work under local anesthesia if the work can not be done in a minute's time which is the duration of time for painless work when face inhaler is used.

Unless one is naturally quick and adept at extraction work it is well not to experiment with gas machines, as the patient is pretty sure to get poor service unless the case is very easy. It seems that the most difficult cases want gas.

Frequently the tooth is extracted and after the patient has returned to consciousness he states that he felt the pain. This is usually due to the fact that the patient was not completely under when the face piece was removed; again it takes eighteen seconds for the final gas inhalation to reach the nerve centers and if one waits a few moments after removing the face piece the best results will be attained.—Geo. C. Cox, *Summary*.

---

Salable and Unsalable Teeth. The time has come in the practice of dentistry when some one should take a firm stand against the ruthless and criminal sacrifice of functionating teeth "on suspicion," and condemn this practice in no uncertain terms. Thousands of teeth have been and are being sacrificed on suspicion that they are the etiological factor in systemic derangement; yet, in a large number of cases, after the loss of such teeth, the patient shows no appreciable improvement. In fact, many patients are in a much more deplorable condition after the loss of their teeth than they were before because of the permanent injury to their masticating apparatus. The loss of a functionating tooth is a serious thing; and until it is absolutely determined that it is impossible to restore infected teeth to such condition that they will no longer be a menace to health, they should not be extracted. That such a procedure is possible can be clearly shown.

The condemnation of all pulpless teeth is the *greatest error ever advocated in dental practice* and falls in the same category as the old "Arthur" method of filing and grinding the teeth out of proximal contact on the theory that teeth not in contact do not decay. Many of the older practitioners here remember seeing the awful condition of mouths so treated.

The advent of the X-ray in dental practice,—while giving us an additional means to be used as a factor in diagnosis, has been a source of danger and a partial cause of the needless sacrifice of many teeth. In the hands of the inexperienced diagnostician, the X-ray picture is the companion-piece to the sword of Damocles, as every shadow shown on the picture is, in these minds, a sword hanging over the head of the patient which may, at any time, fall and cause permanent injury or death.

I am afraid, if many more X-ray equipments are put into the hands of the inexperienced, that the practice of dentistry will degenerate into the extraction of teeth only and the making of artificial dentures. To hear the "hundred per cent." talk, you wonder why the human race has not become extinct from causes due solely to the frightful menace of teeth. Let me warn these radical men that the day is coming, and is not far distant, when an accounting will have to be made for this feature of malpractice,—for such I firmly believe it to be. If we are to make diagnosis using an X-ray picture as the sole means of determining the condition of the suspicious tooth, we are hobbling on a crutch, and a very poor one. On the other hand, I do not want to minimize the importance of a good X-ray picture as a factor in diagnosis, because it is a factor and a most important one; but as a sole means it is decidedly deficient.—T. P. Hinman, *Summary.*

The Significance of "Curable" and "Dead" Teeth.

The dentine gets its vitality from the pulp, but the cementum is nourished by the peridental ligament. Therefore when the pulp is removed only the dentine is affected and the portion of the tooth attached to the cementum is still alive. So there is no such thing in the mouth as a dead or devital tooth, but only a pulpless tooth. The dentist has been responsible for the erroneous idea that teeth from which the pulps have been removed are dead; therefore, our medical friends have taken up the idea that such *dead* teeth should be removed, for any dead thing in the body is necessarily a menace to health. Let us be more careful in our nomenclature and stop calling pulpless teeth "dead" or "devital."

Another diagnosis point of marked importance is the *lamina dura* which lines the socket in the alveolar process and to which is attached the peridental ligament. Particular and careful attention should be given to this for any break in its continuity is viewed with suspicion; and yet there are many teeth which show an apical break,—radio-lucent area,—in which the bone is not affected apically, as this break may be caused by malocclusion due to the tooth in question carrying a crown or filling that is too long. Or, perhaps it is subject to undue stress from being used as an abutment, carrying a bridge pier, the break showing although the tooth may be vital. It must be borne in mind that the X-ray does not show infection but may show the results of infection; that is, bone destruction.

Another structure which may be mentioned is the spongiosum,—that portion of the bone which lies between the lamellae. The tooth, under normal conditions, does not come in contact with this portion of the bone. However, the spongiosum is infiltrated far into its substance by the destruction caused from osteitis of the rarefying type. This type of osteitis, as shown by the X-ray picture, is an indication that the resistance of the patient is below par and the bone is not resisting infection. On the other hand, condensing osteitis, clearly shown by the definite border of condensed bone around the periphery of the radio-lucent area, indicates that the bone is resisting invasion and is setting up a wall to prevent further penetration and destruction. Condensing osteitis is an indication that the resistance of the patient is high and the chances of success in treatment are magnified. In the apical cementum, the same condition prevails; that is, rarefying and condensing cementosis.

Any rarefaction of the cementum is fatal to the tooth, and such tooth should be properly condemned. When the cementum is enlarged by the depositing of successive layers and the root-end is also enlarged, the chances of success through treatment are greatly increased. Nature itself is trying to close the delta of foramina so as to cut off infection, and in fact, does close many of the normal openings leading from the peridental ligament to the pulp. Therefore, the real point in differential diagnosis is the condition of the apical cementum. This condition should be clearly shown in most instances by a well made X-ray picture.

Where the apical cementum has not been rarefied and such apical bone is only partially destroyed and the resistance if the patient is good, the tooth bids fair for treatment. That such teeth can be successfully treated and made no longer a menace to health has many times been clearly proved.—T. P. Hinman, in *Summary*.

---

THE TECHNIC OF PULP CAPPING. If the operation is done upon a tooth that has been aching, upon a pulp that has been exposed by caries where there probably has been a surface abscess, it must be delayed until the abscess has ceased to discharge, and until the inflammation has fully subsided. In such cases the prospect of maintaining vitality will be doubtful; but our success in a few such cases has been such as to justify the effort, especially in the first permanent molars or anterior teeth in very young subjects, the loss of which would seriously impair beauty or derange the occlusion. When the tooth has been quiet under the usual antiseptic sedatives for some days; when there is no tenderness upon percussion; when dressings may be removed without the detection of foul odors, we may proceed with the operation of pulp capping.

The dam should be adjusted before the removal of dressings, and asepsis maintained from start to finish. The preparation of the cavity should be completed, all cuttings removed with the air blast, and the cavity treated with a dressing of sodium carbonate to remove the lactic acid unless this has been done previously, as we prefer. The cavity should be cleansed and dried with alcohol and is now ready for the pulp capping.

For covering the exposed pulp we make a paste of carbolized resin, and the powder of copper cement or any good oxyphosphate cement.

Fletcher's carbolized resin is our vehicle. A small quantity, a few drops of carbolized resin is put out on a clean glass slab, and the oxyphosphate powder is spatulated into it until the mixture forms a soft paste, about the consistency of softening butter. A small quantity of this paste is applied to the point of exposure and pelleted to place with a bit of spunk. When we are sure that we have covered the point of exposure and the thin floor of the cavity with this paste, we proceed to varnish the exposed dentin of the cavity with our antiseptic resin varnish.

Take—Violin Resin ............................ ʒiss

Aristol ............................................ ʒi

Chloroform ................................ f℥i

M.

This will dry sufficiently in a few minutes to receive the cement that is to form the floor of the cavity.

In favorable cases use enough oxyphosphate of copper cement to make a good resistent floor, and finish with a metal filling over this; but in all doubtful cases it will be wise to fill the entire cavity over the pulp capping with the oxyphosphate copper cement and keep the case under observation for a year or so. When satisfied that all is well the surface cement may be cut away, and a metal filling may be inserted.

Our success with this method in favorable cases, has been so uniform that we seldom anticipate failure, and in many of our cases we proceed to make a metal filling at once.

When we have decided to make a metal filling at the same sitting, we introduce only enough of the covering cement to make a solid floor to the cavity, smoothing this with suitable burnishers as it hardens so that it requires little or no trimming. Burnishers may be coated with cocoa butter to keep the soft cement from adhering to them; and the same treatment of the instrument will aid in placing the small quantity of resin paste used to cover the exposure with a very small burnisher coated with cocoa butter. Lift a drop out of the paste, touch it to the point of exposure and push it off with a pellet of punk held in the points of plugging pliers.

A record of all these cases of pulp capping should be kept for future reference, but I regret to say that I have failed in many instances to do this, permitting the press of patients, the hurry of practice, to prevent the performance of this most necessary duty. The record is necessary, not only for the protection of the patient, but for establishing and standardizing the method.

No crown or bridge should be set without first treating the stumps with silver nitrate and then varnishing with the aristol resin varnish before setting with cement. —L. G. Noel, D.D.S., in *Summary*.

